# Validation of a Food Frequency Questionnaire in the Hiroshima/Nagasaki Life Span Study

**DOI:** 10.2188/jea.12.394

**Published:** 2007-11-30

**Authors:** Catherine Sauvaget, Naomi Allen, Mikiko Hayashi, Elizabeth Spencer, Jun Nagano

**Affiliations:** 1 Department of Epidemiology, Radiation Effects Research Foundation, Hiroshima.; 2 Cancer Research UK Epidemiology Unit, Oxford University.; 3 Institute of Health Science, Kyushu University.

**Keywords:** food frequency questionnaire, validity, dietary record, Japan

## Abstract

We evaluated the performance of a 22-item food frequency questionnaire (FFQ) administered in 1980-81 to 3,005 members of the Adult Health Study cohort, part of the Life Span Study. The questionnaire was compared with the records of a 24-hour dietary survey that was performed in 1984-85. From the dietary records, food and nutrient intakes were estimated. The association between the two measures of dietary intake was assessed using Mantel-Haenszel chi-square test and the Spearman’s rank correlation coefficient. The frequency of food intake as measured by the FFQ was linearly associated with food intake as measured by the 24-hour diary, with the exception of dry fish. The highest correlations were observed for beverages, including coffee (0.51), milk (0.32) and black tea (0.26). Foods such as fruit (0.27), confectionery (0.23), rice (0.34) and bread (0.28) were also moderately correlated. These results show that, with the exception of dry fish, the FFQ is moderately correlated with the 24-hour diary and can be used to assess diet intake in this cohort.

The development of methods to measure habitual, long-term dietary intake in epidemiologic studies has been a considerable challenge over the past two decades. The semi-quantitative food frequency questionnaire (FFQ) is the method most commonly used, and usually includes questions on the average frequency of consumption during the past year for a given number of food items. In order to evaluate the performance of a FFQ, dietary intake is often compared with a more objective or detailed measurement, such as biomarkers, an interviewer-assisted 24-hour recall, or self-reported dietary records.

The Radiation Effects Research Foundation in Hiroshima and Nagasaki, Japan is following the Life Span Study cohort, a large cohort of atomic-bomb survivors recruited in 1958.^1^ Several mail surveys have been carried out to examine lifestyle factors among the Life Span Study cohort members^2^^-^^4^ and a self-administered food-frequency questionnaire based on the frequency of consumption of 22 dietary items (FFQ22) was conducted in 1980-81. Since then, two decades have passed and the number of chronic diseases cases among the respondents has become sufficient to allow statistical analysis. Recently, three studies on diet and cancer have been published, based on the questionnaire.^5^^-^^7^ However, the validity of the FFQ22 was not considered in these reports, since no study has been specifically designed to measure its performance. In 1984-85, participants of the Adult Health Study, a sub-cohort of the Life Span Study, were invited to complete a 24-hour dietary record.^8^ The present study aims to compare dietary intake as measured in the FFQ22 with that estimated from the 24-hour dietary record among approximately 3,000 participants of the Adult Health Study.

## METHODS

### Subjects

A self-administered questionnaire including questions on diet (FFQ22), smoking and drinking habits, education, and medical history was sent to all the 55,650 participants of the Life Span Study who were alive as of September 1, 1978.^3^ Responses were received from 40,198 persons (response rate = 72%) between 1980 and 1981. Between 1984 and 1985, 6,743 Adult Health Study members were invited to participate in a 24-hour dietary survey; 6,179 persons (91.6%) accepted and 3,728 persons (60.3%) returned a completed 24-hour diary of food intake. In total, 3,005 participants including 1,133 men (mean age; 60 years old, standard deviation: 11 years, range: 39-89 years old) and 1,872 women (mean age: 57 years old, standard deviation: 7 years, range: 38-80 years old) completed both the FFQ22 and the 24-hour diary, and were available for the present study.

### The FFQ22

Dietary questions consisted of 22 food items: beef and pork, chicken, pork products, dairy products, milk, eggs, fish (except broiled or dry fish), broiled fish, dry fish, salted foods (vegetables and fish gut), green-yellow vegetables (such as pumpkin, carrot, or spinach), fruit, seaweeds, confectionery, tofu, miso soup, rice, bread, non-alcoholic fizzy drinks (such as coca-cola, or soda), black tea, coffee, and green tea. Subjects were asked to state how frequently they ate each of these foods over the past year, based on ‘never’, ‘once or less per day’, ‘twice per day’, and ‘three times or more per day’ for bread and rice, and ‘never’, ‘once or less per day’, ‘two to four times per day’, and ‘five times or more per day’ for green tea. For the remaining foods, the frequency of consumption was ‘never’, ‘once or less per week’, ‘two to four times per week’, and ‘almost everyday’.

### The 24-hour diary

A trained nurse gave detailed instructions for completing the unstructured 24-hour diary and provided participants with examples and a measuring spoon for weighing each food. Each subject chose a day to record his/her usual average meals (breakfast, midmorning snack, lunch, tea, dinner, after-dinner snack), giving names of dishes and foods, and the amount ingested or drunk at each meal during a 24-hour period. Participants were given a prepaid envelope to return the completed diary. A dietician checked and coded the diaries and contacted the subjects if entries needed clarifying. Nutrient consumption was calculated by multiplying the content of each food, derived from the Fourth Edition of the Japan Food Composition Table,^9^ by the frequency of consumption and according to the portion size, as stated in the 24-hour diary.

### Statistical analysis

The food codes from the 24-hour diary were grouped according to the corresponding food items on the FFQ22. The proportion of subjects who recorded whether they had consumed a particular food item in the 24-hour diary was compared with the frequency of consumption as stated in the FFQ22; a test for linear trend was performed using a Mantel-Haenszel chi-square test. The association between the mean intake (g/day) of each food item on the FFQ22 and the 24-hour diary was analyzed using Spearman’s rank correlations. Correlation coefficients were also stratified by sex and age (<60 years, 60+ years). In addition, the mean intake of vitamin C and carotene were estimated from the 24-hour diary data and compared with the intake of green-yellow vegetables and fruit as stated in the FFQ22 as these two nutrients are specific to vegetable and fruit intake.

## RESULTS

Three thousand and five 24-hour diaries were completed and were equally represented throughout the week and the year, with the exception of Sunday which was slightly less represented (8.2%) than the other days (14-15%). Eighty-two percent of the diaries were completed by the subjects themselves, 13% were completed by the spouse and 5% were completed by the daughter or the daughter-in-law.

Table 1 shows the distribution of subjects according to the frequency of consumption as classified by the FFQ22. The majority of subjects reported eating rice twice or more per day and bread once or less per day. More than half reported eating beef/pork, fish and tofu at least 2-4 times per week, and 40-50% ate chicken, pork products, broiled fish, and dry fish once or less per week. Fifty-six percent of subjects consumed dairy products and 37% consumed milk once per week or less, although 75% ate eggs at least 2-4 times per week. Approximately 70-80% of subjects consumed fruit and vegetables at least 2-4 times per week, and nearly half ate salted foods almost daily. Green tea was the most popular beverage, followed by coffee, fizzy drinks and black tea.

**Table 1.** Distribution of food consumption according to the FFQ22 among 3,005 Adult Health Study subjects

	Frequency from the FFQ									

	Never		<= 1/Week		2-4/Week		Almost Daily		Missing	
	n	%	n	%	n	%	n	%	n	%
*Animal Products*										
Beef and Pork	74	2.5	715	23.8	1829	60.9	238	7.9	149	5.0
Chicken	180	6.0	1271	42.3	1159	38.6	67	2.2	328	10.9
Pork Products	424	14.1	1301	43.3	699	23.3	78	2.6	503	16.7
Dairy Products	639	21.3	1041	34.6	446	14.8	259	8.6	620	20.6
Milk	471	15.7	625	20.8	650	21.6	898	29.9	361	12.0
Eggs	52	1.7	542	18.0	1346	44.8	926	30.8	139	4.6
Fish	40	1.3	735	24.5	1708	56.8	391	13.0	131	4.4
Fish (broiled)	121	4.0	1498	49.9	955	31.8	63	2.1	368	12.2
Fish (dry)	514	17.1	1372	45.7	386	12.8	57	1.9	676	22.5

*Vegetables and Fruit*										
Green-yellow Vegetables	28	0.9	695	23.1	1390	46.3	680	22.6	212	7.1
Seaweed	31	1.0	652	21.7	1349	44.9	815	27.1	158	5.3
Fruit	51	1.7	446	14.8	992	33.0	1399	46.6	117	3.9

*Soya Products*										
Tofu	30	1.0	797	26.5	1611	53.6	443	14.7	124	4.1
Miso Soup	102	3.4	615	20.5	943	31.4	1197	39.8	148	4.9

*Beverages*										
Black Tea	1094	36.4	782	26.0	358	11.9	209	7.0	562	18.7
Coffee	638	21.2	657	21.9	517	17.2	884	29.4	309	10.3
Fizzy Drinks	864	28.8	855	28.5	605	20.1	257	8.6	424	14.1
	Never		<= 1/Day		2-4/Day		5+/Day		Missing	
Green Tea	58	1.9	306	10.2	1673	55.7	865	28.8	103	3.4

*Cereal products*	Never		<= 1/Day		2/Day		3+/Day		Missing	
Rice	4	0.1	208	6.9	1520	50.6	1179	39.2	94	3.1
Bread	295	9.8	1769	58.9	72	2.4	4	0.1	865	28.8

*Other Foods*	Never		<= 1/Week		2-4/Week		Almost Daily		Missing	
Salted Foods	198	6.6	494	16.4	654	21.8	1476	49.1	183	6.1
Confectionery	341	11.3	799	26.6	995	33.1	560	18.6	310	10.3

The proportion of subjects who recorded consuming a particular food item in a given 24-hour period according to each FFQ category is shown in Table 2. There was a clear linear association between whether a food was consumed in the diary and increasing frequency of consumption in the FFQ22, with the exception of dry fish and rice. Moreover, the consistency between the dietary measures was generally greatest at a high frequency of consumption; 80-90% of subjects who stated an almost daily intake of eggs, fish, fruit, green-yellow vegetables and salted foods, tofu, miso soup, coffee and rice recorded consuming these foods during a given 24-hour period.

**Table 2.** Proportion of subjects who stated consumption of each food in a given 24-hour according to FFQ category

	Frequency from the FFQ										p-value for linear association^a)^

	Never		<= 1/Week		2-4/Week		Almost Daily		Missing		
	n	%	n	%	n	%	n	%	n	%	
*Animal Products*											
Beef and Pork	24	32.4	314	43.9	1057	57.8	169	71.0	63	42.3	0.001
Chicken	16	8.9	281	22.1	329	28.4	20	29.9	51	15.6	0.001
Pork Products	61	14.4	236	18.1	202	28.9	29	37.2	73	14.5	0.001
Dairy Products	93	14.6	183	17.6	118	26.5	100	38.6	88	14.2	0.001
Milk	127	27.0	230	36.8	302	46.5	617	68.7	112	31.0	0.001
Eggs	28	53.9	358	66.1	964	71.6	772	83.4	88	63.3	0.001
Fish	25	62.5	592	80.5	1450	84.9	354	90.5	101	77.1	0.001
Fish (broiled)	32	26.5	521	34.8	356	37.3	30	47.6	108	29.4	0.005
Fish (dry)	68	13.2	186	13.6	41	10.6	4	7.0	90	13.3	0.588

*Vegetables and Fruit*											
Green-yellow Vegetables	21	75.0	583	83.9	1229	88.4	615	90.4	171	80.7	0.001
Seaweeds	15	48.4	378	58.0	866	64.2	602	73.9	83	52.5	0.001
Fruit	24	47.1	293	65.7	786	79.2	1240	88.6	78	66.7	0.001

*Soya Products*											
Tofu	11	36.7	484	60.7	1094	67.9	356	80.4	72	58.1	0.001
Miso Soup	42	41.2	333	54.2	596	63.2	1014	84.7	87	58.8	0.001

*Beverages*											
Black Tea	39	3.6	67	8.6	55	15.4	78	37.3	32	5.7	0.001
Coffee	104	16.3	243	37.0	304	58.8	718	81.2	71	23.0	0.001
Fizzy Drinks	12	1.4	39	4.6	34	5.6	30	11.7	11	2.6	0.001
	Never		<= 1/Day		2-4/Day		5+/Day		Missing		
Green Tea	30	51.7	182	59.5	1267	75.7	702	81.2	74	71.8	0.001

*Cereal Products*	Never		<= 1/Day		2/Day		3+/Day		Missing				
Rice	3	75.0	202	97.1	1511	99.4	1175	99.7	93	99.0	0.124
Bread	74	25.1	1212	68.5	59	81.9	0	0.0	266	30.8	0.001

*Other Foods*	Never		<= 1/Week		2-4/Week		Almost Daily		Missing		
Salted Foods	102	51.5	337	68.2	455	69.6	1202	81.4	107	58.5	0.001
Confectionery	104	30.5	293	36.7	487	48.9	371	66.3	93	30.0	0.001

a) Based on the Mantel-Haenszel statistics, excluding missing category

The mean intake (g/day) of each food item as estimated from the 24-hour diary according to each food-frequency category of the FFQ22 is shown in Table 3. With the exception of dry fish, the mean intake of each food item increased with increasing food-frequency categories. For example, the mean intake of fruit as estimated from the 24-hour diary increased from 58g/day (which is equivalent to a quarter of an apple or half a banana) in those who recorded they never consumed fruit on the FFQ22, to 189g/day in those who reported eating fruit almost daily. The association between the FFQ22 and the 24-hour diary was strongest for coffee, with a Spearman correlation coefficient of 0.51. Correlations for the other food items ranged between 0.11 for chicken and 0.34 for rice. There was no correlation between the FFQ22 and the 24-hour diary for dry or broiled fish.

**Table 3.** Mean Intake (g/d) and standard deviation (sd) as estimated from the 24-hour diary according to each FFQ category

	Frequency from the FFQ										Correlation Coefficient	p-value for trend^a)^

	Never		<= 1/Week		2-4/Week		Almost Daily		Missing			
	Mean	SD	Mean	SD	Mean	SD	Mean	SD	Mean	SD		
*Animal Products*												
Beef and Pork	12.19	22.78	27.80	46.67	38.62	51.90	51.29	59.09	27.13	46.38	0.17	0.0001
Chicken	4.72	24.00	12.61	32.87	17.39	39.35	17.90	39.61	9.09	28.75	0.11	0.0001
Pork Products	3.04	9.56	4.47	11.78	6.46	12.61	8.71	14.00	3.20	9.94	0.14	0.0001
Dairy Products	1.76	5.41	2.13	5.87	3.45	8.67	5.88	10.70	1.47	4.36	0.17	0.0001
Milk	51.94	101.11	73.87	125.30	91.68	121.00	148.95	137.88	62.55	111.88	0.32	0.0001
Eggs	21.90	31.49	36.57	40.72	38.48	36.45	49.03	35.91	34.91	42.64	0.17	0.0001
Fish	41.06	45.61	60.15	62.44	72.77	70.17	89.28	78.22	56.86	53.98	0.14	0.0001
Fish (broiled)	15.91	31.14	20.83	35.74	22.67	38.82	29.45	36.07	20.10	37.35	0.05	0.0133
Fish (dry)	1.17	5.24	1.06	5.89	1.01	4.97	0.84	3.52	1.52	7.70	-0.03	0.1722

*Vegetables and Fruit*												
Green-yellow Vegetables	27.32	35.79	39.02	45.93	47.91	50.64	56.11	54.68	40.22	48.40	0.14	0.0001
Seaweed	1.79	3.67	5.12	10.35	5.94	11.75	7.32	18.36	4.04	8.67	0.12	0.0001
Fruit	58.40	100.63	103.40	122.52	130.98	130.85	189.26	153.83	127.26	167.54	0.27	0.0001

*Soya Products*												
Tofu	25.87	55.83	52.08	77.21	60.14	78.76	76.20	77.41	47.58	67.34	0.14	0.0001
Miso Soup	8.05	12.00	10.99	12.38	12.65	11.93	17.14	11.35	11.45	11.25	0.25	0.0001

*Beverages*												
Black Tea	6.02	33.70	14.12	50.69	23.72	61.07	70.26	107.77	8.08	34.14	0.26	0.0001
Coffee	9.52	46.07	20.80	60.18	32.22	78.70	75.48	124.31	18.53	60.77	0.51	0.0001
Fizzy Drinks	2.12	18.29	9.10	46.35	11.74	51.32	25.18	75.45	5.37	34.10	0.13	0.0001
	Never		<= 1/Day		2-4/Day		5+/Day		Missing			
Green Tea	147.76	184.00	174.15	203.32	277.01	260.08	404.89	377.68	265.68	283.62	0.23	0.0001

*Cereals Products*	Never		<= 1/Day		2/Day		3+/Day		Missing			
Rice	157.50	127.64	266.80	168.80	327.14	145.42	421.54	168.27	344.93	150.17	0.34	0.0001
Bread	19.75	38.02	53.97	45.59	80.60	51.59	0.00	0.00	26.95	45.55	0.28	0.0001

*Other Foods*	Never		<= 1/Week		2-4/Week		Almost Daily		Missing			
Salted Foods	17.01	24.83	22.40	23.49	24.12	24.81	32.66	27.73	18.81	22.40	0.22	0.0001
Confectionery	17.92	37.35	21.22	39.77	28.71	41.79	42.09	49.63	17.15	36.09	0.23	0.0001

a) Based on Spearman’s rank correlation, excluding missing category

Table 4 presents the correlation coefficients according to sex and age groups. In general, no difference in correlation coefficient was observed between men and women or between the young and the old age groups. However, a slight difference between men and women for beef and pork, fish, seaweed, fruit, salted foods, and confectionery was observed. Also, a difference between the younger and the older groups was noted for eggs, green-yellow vegetables, salted foods, and confectionery.

**Table 4.** Correlation coefficients stratified by sex and age-group

	Sex		Age-group (year)	

	Men	Women	-59	60+
*Animal Products*				
Beef and Pork	0.14	0.21	0.17	0.16
Chicken	0.12	0.12	0.13	0.13
Pork Products	0.16	0.13	0.14	0.10
Dairy Products	0.14	0.16	0.15	0.16
Milk	0.29	0.31	0.31	0.33
Eggs	0.19	0.16	0.15	0.22
Fish	0.17	0.11	0.13	0.14
Fish (broiled)	0.04	0.06	0.04	0.07
Fish (dry)	-0.05	-0.01	-0.01	-0.04

*Vegetables and Fruit*				
Green-yellow Vegetables	0.15	0.13	0.17	0.11
Seaweed	0.18	0.10	0.11	0.16
Fruit	0.27	0.20	0.28	0.24

*Soya Products*				
Tofu	0.14	0.15	0.15	0.14
Miso Soup	0.26	0.23	0.25	0.22

*Beverages*				
Black Tea	0.18	0.22	0.22	0.19
Coffee	0.52	0.48	0.49	0.48
Fizzy Drinks	0.14	0.11	0.11	0.13
Green Tea	0.25	0.20	0.23	0.21

*Cereals Products*				
Rice	0.29	0.30	0.33	0.30
Bread	0.32	0.31	0.33	0.33

*Other Foods*				
Salted Foods	0.26	0.20	0.26	0.19
Confectionery	0.15	0.23	0.26	0.21

The daily mean intake of carotene and vitamin C as estimated from the 24-hour diary according to the frequency of consumption of green-yellow vegetables and fruit as stated on the FFQ22 is shown in Table 5. The mean intake of carotene was moderately correlated with green-yellow vegetables intake, with a Spearman correlation coefficient of 0.16 for carotene from all vegetables and 0.12 for carotene derived from green-yellow vegetables. Increasing fruit consumption was also moderately correlated with estimated carotene intake derived from fruit and also with vitamin C intake, with correlation coefficients of 0.20 and 0.22 for fruit-derived carotene and vitamin C, respectively.

**Table 5.** Mean intake and standard deviation (SD) of carotene and vitamin C as estimated from the 24-hour diary according to vegetable and fruit intake on the FFQ22

Mean intake from the 24-hour diary	Frequency from the FFQ									

	Never		<= 1/Week		2-4/Week		Almost Daily		Correlation Coefficient	Test for linear trend
	Mean	SD	Mean	SD	Mean	SD	Mean	SD		
*Green-yellow vegetables*										
Carotene from all vegetables (*µ*g/d)	771.92	1050.71	1358.00	1511.04	1743.42	1814.96	2006.28	1911.73	0.16	0.0001
Carotene from green-yellow vegetables (*µ*g/d)	455.04	959.82	684.71	1220.47	926.58	1489.06	1032.26	1418.56	0.12	0.0001
*Fruit*										
Carotene from fruit (*µ*g/d)	40.67	80.91	89.78	200.97	109.64	215.22	151.95	255.95	0.20	0.0001
Vitamin C from fruit (mg/d)	41.57	27.30	44.99	30.49	51.05	32.54	63.17	38.61	0.22	0.0001

## DISCUSSION

This study has compared the performance of the FFQ22, a food frequency questionnaire used among Life Span Study participants between 1980 and 1981, with a 24-hour diary survey performed in 1984. There was a linear association between the frequency of consumption as stated on the FFQ22 and the 24-hour diary for all foods, with the exception of dry fish and rice. In addition, the mean food intake as estimated from the 24-hour diary was linearly associated with increasing frequency of intake as stated on the FFQ22 for all foods, except dry fish. Further, the linear association between the mean carotene and vitamin C intake in relation to green-yellow vegetables and fruit consumption suggests that the FFQ22 can be used as a valid tool in which to measure dietary intake in this population. Dry fish was the only food item on the FFQ22 that was not associated with the 24-hour diary, most likely a reflection of its low consumption in this population, and should therefore be used with caution.

Although some participants who stated that they never consumed food items (such as fish, green-yellow vegetables and rice), actually recorded the food in the 24-hour diary; they represented only about 1% for fish and vegetables, and 0.1% for rice (see Table2). Furthermore, the “never” consumers had a low intake as estimated from the 24-hour diary. For example, the mean intake of pork products in the “never” category representing one fifth of a bacon slice or sausage; 22g of egg correspond to less than a half of a hen egg; and 58g of fruit is equivalent to a quarter of an apple, or half a banana. With regard to those with a missing frequency of a given food in the FFQ22, their proportion of consumption (Table2) and their mean intake (Table3) were similar to the values of those who answered “never” or “once or less per week or per day”. However, the missing values for green tea and rice were comparable to the “2-4 times per day” category, but these subjects with missing data represented only 3% of the study population. It is therefore likely that the participants did not report the intake frequency of a food because they never or scarcely ate this food. For future research based on the present FFQ22, the “never” and possibly “missing” category would be better grouped with the “once or less per week”, or “once or less per day” category.

The administration of a FFQ is particularly relevant in large prospective studies since it may be self-administered, it is easy to use, and is relatively inexpensive to process.^10^ However, FFQs are based on a limited number of food items and rely heavily on the respondents’ memory and their interpretation of the questions. A more direct method, such as a 24-hour dietary record, is much more accurate since the intake of foods is recorded in real time and without restrictions, and allows a direct estimation of the portion size and nutrient intake.^11^ However, such a method increases the burden on study participants and requires substantially more resources for data management. As there is no gold standard in measuring habitual dietary intakes,^12^ direct record methods are often used on a small number of subjects to obtain referent data for validating a less accurate method such as a FFQ.^13^^-^^15^

The present study was performed on a large number of subjects and, although we observed modest correlation coefficients for most foods, they are of a similar magnitude to those found in other studies, which are often between 0.2 and 0.4, and which are unlikely to be higher than 0.5-0.6.^16^ Correlation coefficients greater than 0.2 were found for rice, bread, milk, fruit, salted foods, miso soup, confectionery, black tea, coffee, and green tea. This may partly be because these items are more easily measured,^17^ as they are usually served as one unit, and are often consumed separately from other foodstuffs.^18^ Indeed, a previous validation study performed in Japan showed that food products included in mixed dishes, like meat or vegetables, had lower correlation coefficients.^19^ In addition, the foods that are eaten more frequently are better estimated than foods eaten less frequently.^20^

However, our reference method presents some limitations that may have attenuated the observed correlations with the FFQ22. Firstly, the diaries were recorded only for a 24-hour period and may therefore not capture day-to-day variation in food intake,^21^ leading to possible underestimation of the association.^22^ A recent validation study of a FFQ among a Japanese population, based on four 4-day diary records showed higher correlation coefficients than our study.^19^ In addition, the 24-hour diaries were completed between three and four years after the FFQ22, thus increasing the possibility that an individual’s dietary habit may have changed. However, on a population level, an adult’s dietary pattern is thought to remain fairly stable over time,^10^ and national food consumption and food availability patterns in Japan were relatively constant during the period between the two surveys (1980 and 1984).^23^

In conclusion, all food items as measured by the FFQ were linearly associated with food intake as measured by the 24-hour diary, with the exception of dry fish. These findings suggest that the FFQ22 can be used to measure habitual dietary intake in the Life Span Study and supplement the previous reports based on this questionnaire.^5^^-^^7^
